# Sustainable diets: where from and where to?

**DOI:** 10.1017/jns.2025.10049

**Published:** 2025-11-03

**Authors:** Lesley Macheka, Rebecca Kanter, Mark Lawrence, Sandro Dernini, Farah Naja, Stineke Oenema

**Affiliations:** 1 Department of Food Processing Technology, Marondera University of Agricultural Sciences and Technology, Marondera, Zimbabwe; 2 Department of Nutrition, Faculty of Medicine, University of Chile, Santiago, Chile; 3 School of Exercise and Nutrition Sciences, Institute for Physical Activity and Nutrition, Deakin University, Melbourne, Australia; 4 Forum on Mediterranean Food Cultures, Rome, Italy; 5 Department of Clinical Nutrition and Dietetics, College of Health Sciences, University of Sharjah, Sharjah, United Arab Emirates; 6 Faculty of Agriculture and Food Sciences, American University of Beirut, Beirut, Lebanon; 7 Chair of the IUNS Sustainable Diets Task Force, Den Haag, Netherlands

**Keywords:** Environmental sustainability, Food systems, Indigenous foods, Sustainable diets, AIVs, African Indigenous Vegetables, aHEI, Alternate Healthy Eating Index, CIHEAM, International Centre for Advanced Mediterranean Agronomic Studies, COVID-19, Coronavirus Disease 2019, DASH, Dietary Approaches to Stop Hypertension, EPIS, Exploration, Preparation, Implementation, Sustainment (Framework), FAO, Food and Agriculture Organization of the United Nations, FBDGs, Food-Based Dietary Guidelines, GHG, Greenhouse Gas, HSR, Health Star Rating (System), IFMeD, International Foundation of Mediterranean Diet, LMD, Lebanese Mediterranean Diet, LMICs, Low- and Middle-Income Countries, SDGs, Sustainable Development Goals, SFS, Sustainable Food System, SHARP, Sustainable, Healthy, Affordable, Reliable, and Preferable (Diet Model), TLD, Traditional Lebanese Diet, UNESCO, United Nations Educational, Scientific and Cultural Organization, UPF, Ultra-Processed Foods, WHO, World Health Organization

## Abstract

The multilevel dimensions of sustainable diets associating food systems, public health, environmental sustainability, and culture are presented in this paper. It begins by defining sustainable diets as those that are healthful, have low environmental impacts, are affordable, and culturally acceptable. The discussion includes the history of research on sustainable diets, from initial studies focused on environmental impacts to more recent, comprehensive frameworks that integrate affordability, cultural relevance, and nutritional adequacy as key dimensions of diet sustainability. In addition, the paper highlights recent innovations, such as the Planetary Health Diet of EAT–Lancet and the SHARP model, and the conflicts and optimum trade-offs between sustainability and nutrition, particularly within low- and middle-income countries. Case descriptions of Mediterranean Diet with a focus on Traditional Lebanese Diet, and African Indigenous Foods demonstrate culturally confined dietary patterns associated with sustainability objectives. These examples show that sustainable diets are not a single set of prescriptions, but a series of multiple pathways that are shaped by local food environments, ecological belts, and sociocultural heritages. The paper also describes major policy and governance activities necessary to promote sustainable diets. Finally, the paper addresses measurement challenges and advocates for better indicator options to measure sustainable food systems in all their facets and for participatory and context-specific approaches. The discussion concludes that fairer and culturally diverse inclusion strategies, system change, and political determination are imperative in achieving sustainable diets. Diets able to sustain are posited as agents capable of driving the 2030 agenda, enhancing planetary health and social integrity.

## Introduction

In 2010, a consensus definition for sustainable diets was reached at the International Scientific Symposium on Sustainable Diets and Biodiversity: United against hunger, organised by Food and Agriculture Organization (FAO) and Bioversity International, as ‘*those diets with low environmental impacts which contribute to food and nutrition security and to healthy life for present and future generations. Sustainable diets are protective and respectful of biodiversity and ecosystems, culturally acceptable, accessible, economically fair and affordable; nutritionally adequate, safe and healthy; while optimizing natural and human resources’*.^(^ ^1^ ^)^ Sustainable diets and sustainable food systems (SFS) are intrinsically linked through dynamic, multidirectional interactions that influence environmental health, human nutrition, and socioeconomic development.^(^ ^2^ ^)^ In 2019, FAO and the World Health Organization (WHO) defined sustainable healthy diets as those that ‘promote all dimensions of individuals’ health and well-being; have low environmental pressure and impact; are accessible, affordable, safe and equitable; and are culturally acceptable’.^(^ ^3^ ^)^ A SFS is defined as a food system that ensures food and nutrition security for all in such a way that the economic, social, and environmental bases to generate food and nutrition security of future generations are not compromised, while a food system gathers all the elements (environment, people, inputs, processes, infrastructures, institutions, etc.) and activities that relate to the production, processing, distribution, preparation and consumption of food, and the outputs of these activities, including socio-economic and environmental outcomes.^(^ ^4^ ^)^ The translation of this definition into practice requires systemic and coordinated shifts in food systems, encompassing food production, processing, distribution, consumption, and waste management.

Food systems exert profound influences on dietary choices through availability, affordability, convenience, and marketing of foods.^(^ ^5^ ^)^ Industrialised food systems, which dominate global markets, have been characterised by high levels of resource input, monoculture production, and long supply chains. These systems are characterised by the supply and consumption of ultra-processed foods that are associated with adverse health outcomes (1) and greenhouse gas (GHG) emissions, deforestation, biodiversity loss, novel entities such as plastic and industrial chemicals in waste streams, and water scarcity.^(^ ^6^ ^–^ ^11^ ^)^ As such, reorienting food systems towards sustainability involves promoting agroecological production, reducing food waste, enhancing local value chains, and facilitating dietary transitions towards more plant-based, minimally processed foods.^(^ ^12^ ^)^ Dietary patterns based on diversified plant-based foods such as fruits, vegetables, legumes, nuts, and whole grains have been shown to be environmentally less intensive than diets rich in animal-source foods. Studies by Poore and Nemecek^(^ ^13^ ^)^ revealed that animal products contribute 83% of GHG emissions from the average diet while providing only 18% of calories. Moreover, water and land use associated with animal agriculture is significantly higher than that required for plant-based diets. Therefore, shifting consumption away from red meat and dairy towards more sustainable protein sources can substantially lower environmental burdens.^(^ ^14^ ^)^

However, environmental sustainability should not compromise nutrition security. Livestock, particularly in low-income settings, contributes to micronutrient adequacy (such as vitamin B12, iron, and zinc) and income generation. Therefore, sustainability strategies must be context-specific, acknowledging trade-offs and co-benefits between environmental integrity, nutritional adequacy, and livelihoods.^(^ ^15^ ^)^ Additionally, sustainable diets should resonate with local culinary traditions and social norms to ensure adoption and equity, especially among marginalised populations.^(^ ^16^ ^)^ Moreso, food systems transformation entails innovations in food environments and value chains. According to Herforth *et al.* ^(^ ^16^ ^)^, urban food environments play a critical role in shaping sustainable diets through food retail, marketing, and policy interventions such as fiscal measures and labelling schemes. Similarly, short food supply chains and territorial markets promote local biodiversity, reduce emissions from transport, and improve income for smallholder farmers. Agroecological approaches, circular bioeconomy principles, and integration of traditional knowledge can enhance food systems’ resilience to climate and economic shocks.

Overall, sustainable diets are both outcomes of and catalysts for SFS, with dietary transitions playing a pivotal role in driving systemic transformation.^(^ ^17^ ^)^ The nexus among environmental health, nutrition, and social equity requires integrated strategies to accommodate ecological limits and human needs. Ensuring this balance requires an inter-sectoral approach, in which different stakeholders from along the food chain can come together to form a strong political commitment.

## Scientific advances in sustainable diets

Scientific advances in sustainable diets have increasingly bridged the gap between nutrition, environmental sustainability, and the socio-cultural dimensions of food systems. Initially, framed as an ethical and ecological concern, sustainable diets are now the subject of interdisciplinary research, policy advocacy, and methodological innovation. One major scientific advance has been the integration of environmental impact assessments into dietary studies. Poore and Nemecek^(^ ^13^ ^)^ conducted life cycle assessment of global food production, revealing that food accounts for over 26% of global GHG emissions, with livestock production alone responsible for nearly 60% of these emissions. This study demonstrated that shifting dietary patterns towards plant-based foods could reduce food-related GHG emissions by up to 49%, water use by 19%, and land use by 76%, underscoring the environmental imperative of sustainable diets. Willett *et al.* ^(^ ^12^ ^)^ advanced this research by proposing a planetary health diet (i.e, the ‘EAT Lancet diet’), a flexible reference model largely based on vegetables, fruits, whole grains, legumes, nuts, and unsaturated oils, with low to moderate consumption of fish and poultry, and limited red meat, dairy, and added sugars. Further scientific advances have emerged in the methodological assessment of sustainable diets. While initial research focused heavily on environmental dimensions, such as carbon footprint, land use, and eutrophication, recent studies have developed multidimensional indices that include nutritional adequacy, cultural acceptability, and affordability. For example, the Sustainable Diet Index^(^ ^18^ ^)^ and the SHARP diet model (Sustainable, Healthy, Affordable, Reliable, and Preferable)^(^ ^19^ ^,^ ^20^ ^)^ allow for comparative assessment of dietary scenarios across diverse populations.

Furthermore, nutritional science has also contributed to the refinement of sustainable diets. Emerging evidence suggests that diversified plant-based diets can meet protein and micronutrient requirements if carefully planned. However, studies caution against overly restrictive vegan diets in regions where deficiencies in vitamin B12, iron, and calcium are prevalent.^(^ ^21^ ^)^ In these contexts, moderate consumption of animal-source foods, especially dairy, eggs, and fish, may be essential for vulnerable groups such as children, pregnant women, and the elderly. As such, sustainability must be balanced with nutritional adequacy and local food access. Additionally, advancements in behavioural and cultural research have shed light on the acceptability and feasibility of sustainable diets. Studies by Perignon *et al.* ^(^ ^21^ ^)^ revealed that food choices are shaped not only by health and environmental awareness but also by taste preferences, cultural norms, gender roles, and religious taboos. Therefore, interventions must be context-specific and culturally accepted. For example, insights from participatory research by Fanzo *et al.* ^(^ ^15^ ^)^ in rural African and South Asian communities indicated that traditional food practices can be aligned with sustainable diet goals, particularly when coupled with nutrition education and community engagement.

Moreover, urban food environments have become a focal point in sustainable diet research. The proliferation of ultra-processed foods, defined by the NOVA classification as industrial formulations with little or no intact foods, typically containing additives, flavourings, colourings, preservatives, and other industrial ingredients, e.g., sugary beverages, packaged snacks, instant noodles, and reconstituted meat products,^(^ ^22^ ^–^ ^24^ ^)^ in urban areas of both the Global North and South has been linked to rising obesity rates and non-communicable diseases^(^ ^25^ ^,^ ^26^ ^)^ while simultaneously contributing to environmental degradation^(^ ^27^ ^,^ ^28^ ^)^ through high resource use and waste generation. Scientific consensus now supports policy tools such as front-of-pack labelling, taxation on sugary beverages, and food marketing restrictions to guide consumers towards healthier and more sustainable food choices.^(^ ^29^ ^)^ Building on these nutritional and behavioural insights, systems thinking offers a framework to address the structural and interconnected factors that shape dietary patterns.^(^ ^30^ ^)^ Systems thinking shifts the focus from isolated interventions to coordinated, multi-sectoral strategies that leverage synergies across health, environmental, and socio-economic objectives.^(^ ^31^ ^)^

In summary, scientific advances in sustainability science and systems thinking have elevated the importance of food systems transformation.^(^ ^3^ ^)^ Instead of addressing individual behaviour in isolation, researchers now model food environments, supply chains, and governance structures to understand how systemic interventions, such as agroecology, territorial markets, and public procurement, can support sustainable diets. Approaches including circular food economies, plant-based meat replacements, and climate-resilient crops present promising options, but scalability and equity are key research needs. Significant strides have been made; however, future research should integrate dimensions such as affordability, food sovereignty, and gender equity. With sustainable diets on the rise worldwide, there is a need for them to be evidence-informed, contextually appropriate, and culturally acceptable to ensure the long-term sustainability of both health and ecological benefits.

## Why measure sustainability in diets?

Sustainability in diets is a multidimensional concept that connects what people eat to both human and planetary health. In recent years, some countries have started to reflect this in their Food-Based Dietary Guidelines (FBDGs). For example, Sweden and Brazil have integrated environmental sustainability into their national guidelines, recognising that food choices not only shape nutrition and health but also influence ecosystems and natural resources.^(^ ^32^ ^,^ ^33^ ^)^ However, despite these promising examples, the integration of environmental considerations remains uneven globally, with many national FBDGs still chiefly prioritise nutrient adequacy, omitting essential planetary dimensions such as GHG emissions, biodiversity degradation, and water use such integration.^(^ ^32^ ^,^ ^34^ ^)^ Moving forward, it is critical that emerging evidence on environmental footprints of diets (e.g., water use, GHG emissions, and biodiversity impact) is systematically incorporated into dietary guidance to ensure alignment between human health and planetary health. However, there are still many studies that consider, and thus, measure sustainability in diets based on a single concept, or dimension of the previous definition, such as environmental impacts.^(^ ^35^ ^)^ A review of FBDGs identified countries with government-endorsed FBDGs that made explicit mention of environmental sustainability. As climate change continues to impact food systems, and more FBDGs consider the sustainability of diets and food systems, the measurement of sustainability in diets has an even greater importance and relevance, especially for public health nutrition practice. It is important to measure sustainability in diets to better design interventions to improve, monitor and evaluate changes in diets and related food systems, and develop strategies for implementation and dissemination regarding sustainable food options. And understand the elements to improve food systems to align with FBDGs.

### Intervention development

The purpose of measuring the sustainability of diets for public health nutrition practice is to shift the study focus away from specific individual diets to that of planetary health to improve public health nutrition outcomes. For example, it is useful to identify which traditional culinary preparations are still consumed and liked in a population that can be subsequently used for nutrition intervention development related to healthy and sustainable diets. Ethnographic approaches are particularly valuable in this regard, as they uncover cultural meanings, taboos, and practices that shape food choices, thereby ensuring that interventions resonate with communities.^(^ ^36^ ^)^ Structured manuals, such as the Campaign for Rural England’s Mapping Local Food Webs Guide,^(^ ^37^ ^)^ provide systematic frameworks for participatory research, while research guidelines establish principles for methodological rigour. These manuals should, however, be complemented by other sources of data, such as national food balance sheets, agricultural harvest statistics, and dietary surveys, to ensure that interventions are both culturally relevant and evidence based.^(^ ^38^ ^)^ By triangulating ethnographic insights with quantitative data, policymakers and practitioners can design culturally competent, sustainable programmes, as demonstrated in contexts where sustainability principles have been integrated into national FBGDs, such as in Mexico.^(^ ^39^ ^,^ ^40^ ^)^

However, intervention development should not only focus on designing culturally relevant strategies but must also consider the realities of people’s everyday food choices and the environments in which they live. For example, in many low- and middle-income settings, diets are often based on starchy staples, with limited access to diverse food groups or high dependence on animal-sourced foods.^(^ ^41^ ^)^ Additionally, local environmental conditions such as water availability, soil fertility, and biodiversity also shape what foods are produced and consumed.^(^ ^42^ ^)^ Moreover, socio-economic realities (including income distribution, food affordability, and informal markets) can influence adoption of dietary recommendations.^(^ ^43^ ^)^ By grounding interventions in both culture and context, programs can more effectively balance sustainability goals with feasibility and acceptability at the community level.

### Monitoring and evaluation

Describing multiple aspects of sustainable diets can help identify what to monitor and subsequently, evaluate as part of public health nutrition programs. Monitoring should focus on realistic proxy indicators that reflect both dietary behaviours and food system dynamics. These include the roles of food system actors involved in the provision of fresh and seasonal products (e.g., farmers, local markets, and retailers), the physical and economic accessibility of culturally relevant and sustainable culinary preparations, and evidence of their consumption at household or community levels. For instance, changes in dietary habits, such as shifts towards more plant-based meals, can be assessed not only in terms of nutrient adequacy but also in relation to environmental impacts like GHG emissions and land use.^(^ ^12^ ^)^ Moreover, crises such as droughts, floods, or pandemics may trigger local food system resilience strategies, which can serve as additional indicators of adaptation and sustainability.^(^ ^44^ ^)^ Sustainable diets can therefore be monitored as potential indicators of change, particularly when social norms and agricultural practices evolve simultaneously.

Some of the most notable sustainable diet measurement studies related to monitoring and evaluation, come from using the EAT-Lancet diet as a reference.^(^ ^44^ ^,^ ^45^ ^)^ While long-term improvements in human and planetary health are the ultimate goals, monitoring and evaluation frameworks must rely on intermediate, measurable dimensions. These metrics may include human health outcomes (such as obesity prevalence, micronutrient adequacy indices, and incidence of diet-related non-communicable diseases), environmental sustainability indicators (including GHG emissions, land use, and water footprint), and integrated indices that combine nutrition, environment, and affordability. Such multidimensional metrics ensure that monitoring frameworks capture the full complexity of sustainable diets. The EAT-Lancet 2.0 advances this work to promote securing a just transition to healthy, environmentally sustainable diets for all.^(^ ^46^ ^)^

### Implementation and dissemination

Studies of sustainable diets and dietary practices are imperative for the development of effective strategies for the long-term feasibility of interventions and their sound dissemination. Feasibility includes figuring out in which institutions, often across different food systems sectors, to base a public health nutrition intervention, how to institutionalise the intervention within a particular setting, and perform capacity building, among other actions outlined in the Preparation, Implementation, Sustainment (EPIS) framework.^(^ ^47^ ^)^ It follows that more culturally adapted public health nutrition strategies are more feasible as they are more likely to be acceptable and resonant within local settings.^(^ ^48^ ^)^

### How to measure sustainable diets

Often sustainability in diets has been measured or assessed through only one dimension or aspect. Tiboni-Oschilewski *et al.* ^(^ ^49^ ^)^ summarise the main methods that have been used for the study of sustainable diets from one dimension; while emphasising the need for more research studies that consider multiple dimensions in the measurement of sustainable dietary patterns.^(^ ^49^ ^)^ In their paper, they describe in detail the use of different methods and diverse data sources to measure sustainable diets. As the sociocultural dimension of sustainable diets is often the hardest to assess, prior to any data collection, a comprehensive literature search and related formative research about the food culture under study is recommended to provide an initial knowledge base for the researcher or observer.^(^ ^50^ ^)^

## Case studies on sustainable diets

This section presents case studies that demonstrate locally adapted strategies for promoting sustainable diets. The included case studies highlight efforts to integrate indigenous foods, reduce environmental footprints, improve nutrition, and support livelihoods, particularly among vulnerable populations. While global frameworks and dietary models such as the EAT–Lancet reference diet^(^ ^12^ ^)^ provide a general direction for sustainable diets, translating these into real-world practices requires contextual evidence. Case studies are essential for illustrating how diverse communities and food systems operationalise sustainable diets in response to cultural norms, resource constraints, environmental challenges, and public health priorities. As the international community advocates for food system transformation,^(^ ^51^ ^)^ these real-world experiences provide critical evidence for designing inclusive, scalable, and context-specific solutions that align with both planetary boundaries and human health needs.

### The Mediterranean diet as a case study model

The incorporation of sustainability into dietary guidelines has gained increasing attention over recent decades, aiming to make diets healthier for consumers as well as for the environment. After the publication of the first dietary guidelines for sustainability by Gussow and Clancy^(^ ^52^ ^)^, criticisms have continued to ignite^(^ ^1^ ^,^ ^53^ ^)^ controversial debates on definitions and assessments among ‘sustainable diets’,^(^ ^1^ ^)^ ‘sustainable healthy diets’,^(^ ^3^ ^)^ and ‘healthy diets’^(^ ^54^ ^)^ in the context of a SFSs transformation, which is still an ongoing debate.

Within this international debate on the sustainability of diets and food systems, the Mediterranean diet, predominantly a plant-based diet with low consumption of animal and industrial products, has been increasingly studied as a model of a sustainable diet, context-specific for the Mediterranean, and acknowledged by the United Nations Educational, Scientific and Cultural Organization (UNESCO) as an intangible cultural heritage of humankind under risk of erosion.^(^ ^55^ ^)^ The initial scientific interest in the Mediterranean diet was driven by its health-promoting characteristics, particularly its protective effect against cardiovascular disease and non-communicable diseases. Supported by epidemiological and clinical research,^(^ ^56^ ^)^ it quickly became established as a benchmark for healthy eating patterns. Over the last two decades, additional studies have expanded the perspective to highlight its multiple interdependent environmental, socio-cultural, and economic sustainability,^(^ ^53^ ^)^ showing that its predominantly plant-based structure, biodiversity support, and cultural traditions contribute also to lower ecological footprints.^(^ ^40^ ^)^ This evolution underscores the Mediterranean diet’s dual role as both a health-dietary pattern as well as a sustainable diets model.^(^ ^55^ ^)^ Throughout the Mediterranean region, adherence to the Mediterranean diet is decreasing especially among young people. The Mediterranean diet is a widely accepted healthy diet supported by scientific evidence from epidemiology and clinical trials, including randomised control trials, and systematic reviews and meta-analyses. The Mediterranean diet dietary pattern with proven health benefits, especially regarding the prevention of non-communicable diseases at a time when their incidence is increasing worldwide.^(^ ^57^ ^)^ It has also been recognised as a sustainable diet model with multiple interdependent benefits.^(^ ^58^ ^)^

Throughout the Mediterranean region, the adherence to the Mediterranean diet is decreasing especially among young people, with proliferation of ultra-processed foods and negative accompanying effects on human health. Scientific evidence shows a tendency of Mediterranean populations to change their dietary patterns in favour of unhealthy dietary patterns and mainly among the young generation. Some Southern and Eastern Mediterranean countries are still experiencing the ‘nutritional transition’, in which problems of under-nutrition coexist with overweight, obesity, and diet-related chronic diseases.^(^ ^59^ ^)^ The concept of the Mediterranean diet has undergone a progressive evolution, from a healthy dietary model to a sustainable dietary model,^(^ ^60^ ^)^ a lifestyle in continuous evolution, closely related through time to the particular historic and geographic mosaic that is the Mediterranean. Studies on the Mediterranean diet were/are mainly focused on health/nutrition impacts of its characteristic foods, while the importance of its cultural, social, and economic food dimensions is not taken fully into account on the ground, while it is shared by all Mediterranean people, beyond a simple physiological need for energy and health. In 2015, to address the growing erosion of the heritage of the Mediterranean Diet, due to the continued loss of its adherence by Mediterranean populations, a new multidimensional conceptual framework ‘the Med Diet 4.0’ was designed to highlight its multiples sustainable benefits, with country-specific variations, with the strategic purpose to attract a broader spectrum of interested stakeholders from other sectors.^(^ ^60^ ^)^ In the design of the Med Diet 4.0, country-specific variations and interdependences were highlighted to be adapted to different Med contexts, with four interdependent sustainable benefits of the Mediterranean diet characterised in parallel:

1. *Well-documented health and nutrition benefits.* ^(^ ^61^ ^)^ The Mediterranean diet was cited, in the EAT-Lancet Commission report on diet in the Anthropocene, as the most studied example of a healthy diet.^(^ ^12^ ^)^
2. *Low environmental impacts and richness in biodiversity,* essentially a plant-based diet with low consumption of animal products and a type of production that has less impact on natural resources than other diets.^(^ ^62^ ^,^ ^63^ ^)^
3. *Positive local economic returns*, creation of new jobs, reduction of rural poverty and migration^(^ ^64^ ^)^
4. *High socio-cultural food values,* dialogue among different cultural identities and food traditions, mutual respect, and social inclusion.^(^ ^65^ ^)^ Recognised by UNESCO as an «Intangible Cultural Heritage of Humanity».^(^ ^55^ ^)^

As a case study for sustainable diets, the Mediterranean diet started to be investigated in 2011, through a dedicated international workshop on *Guidelines for the Sustainability of the Mediterranean Diet,* organised by FAO and International Centre for Advanced Mediterranean Agronomic Studies (CIHEAM), to assess the sustainability of the Mediterranean diet as a case study.^(^ ^66^ ^)^ This led to a consensus proposal for the identification of key nutritional indicators for the Mediterranean diet as a sustainable diet case study.^(^ ^67^ ^)^ The first world Mediterranean diet conference on ‘*The Revitalizing the Mediterranean Diet: from a healthy dietary pattern to a healthy Mediterranean sustainable lifestyle’* was organised in 2016 in Milan (IFMeD, 2016), with a session moderated by FENS (Federation of European Nutrition Societies) focused on *‘The Challenge of mainstreaming the sustainability of the Mediterranean diet within Mediterranean national dietary guidelines’*. As an outcome an updated Mediterranean diet cultural framework, with frugality focused on its environmental benefits, was developed,^(^ ^68^ ^)^ followed in 2017 by an international workshop on *Development of voluntary guidelines for the sustainability of the Mediterranean diet in the Mediterranean region*, where common principles of the Mediterranean diet were highlighted and incorporated, such as: Variety and balanced food combination: Different food, with more fruits and vegetables of diverse colors; Seasonality: Fresh foods, minimally processed; Traditional, local food products, biodiversity, agro-eco-friendliness: Territorial linkages—sustainable rural development; Culinary activities: Preservation and transmission of food knowledge, skills, practices, and heritage and pleasure of eating; Conviviality: Pleasure of eating together—dialogues between people and cultures; Frugality and moderation: Small portion sizes—major public health challenge of obesity—food has value, do not waste; Active living: Physical activity—non-sedentary lifestyle.

In 2023, in continuation of this consensus process, as outcome of the Third World Mediterranean Diet Conference on ‘C*hange of route towards more sustainable and resilient food systems in the Mediterranean countries: The Mediterranean diet as a strategic resource for accelerating the Agenda 2030 in the Region*’ organised at the CIHEAM Bari, a Joint Med Diet Task Force of CIHEAM, FENS, and IUNS was formed to set the path for reversing the erosion of the Mediterranean diet heritage, by promoting its benefits, as a way of living. The rationale for co-developing this joint collaborative effort was based on the recognition that the challenges threatening the adherence to the Mediterranean diet as a sustainable healthy diet are complex, interrelated, and context specific. The traditional ways of consuming and producing food in the Mediterranean area have changed considerably, due to economic, social, cultural, demographic, and technological trends, increasing globalisation, urbanisation, and shifting lifestyles with increased sedentary daily life. Therefore, a SFS approach, context-specific, is required for a sustainable revitalisation of the Mediterranean diet, grounded in multi-stakeholder collaborations and consensus, rather than fragmented sector-specific solutions.

The assessment of the sustainability of the Mediterranean diet is a challenge of complexity for its multiple dimensions and nutritional, environmental, economic, and socio-cultural interconnected impacts, with med country-specific variations. All of these impacts are interdependent so that changes in one can led to changes in others. The challenge is to fully take into account this systemic complexity without breaking it down into its different parts, which would cause it to lose its interactive characteristics. Reviews of indicators used to evaluate the sustainability of each dimension of the Mediterranean diet show that there was no uniformity in their assessments, mostly focused to health and environmental impacts, and less on socio-cultural and economic dimensions, often weak, fragmented and arbitrary, mainly quantitative and less qualitative.^(^ ^69^ ^)^

### Mediterranean diet as a prototype for healthy sustainable diets: the case of the Lebanese traditional diet

In the last decade, within the SFS transformation process, the Mediterranean diet has been increasingly acknowledged as a sustainable diet model. As such, several countries, especially Mediterranean countries, became increasingly invested in examining the health benefits and the sustainability of their traditional dietary intake. In this section, the case of the Traditional Lebanese Diet (TLD) as a version of a sustainable Mediterranean diet is presented. Lebanon is a small middle-income country on the Eastern shore of the Mediterranean Sea. The three main aspects of this case study include 1) the characterisation of the TLD and its health benefits 2), the presentation of the TLD as a version of the Mediterranean diet from the east side of the Mediterranean basin, and 3) lastly its sustainability in terms of environmental footprints, social aspects, and cost.

Several research studies have examined the prevalent dietary patterns in Lebanon among adults and among adolescents and children.^(^ ^70^ ^–^ ^73^ ^)^ The number of data-driven patterns identified by these studies varied between 2 and 4 patterns, mainly a traditional Lebanese and a Western type. The TLD consisted mainly of fruits, vegetables, legumes olives, burghul (crushed wheat), whole milk and dairy products, starchy vegetables, eggs, dried fruits, in addition to olives and olive oil.^(^ ^70^ ^)^ On the other hand, the Western type of diet included processed meat, red meat, refined grains, sugar sweetened beverages and sweets. These studies documented a positive association between TLD and several health benefits. For instance, a one unit increase in the score of the TLD was found to be associated with 54% lower odds of type 2 diabetes among Lebanese adults.^(^ ^71^ ^,^ ^74^ ^)^

Similarly, another study examining metabolic health among obese adults, aged 18 years and older, showed that subjects with higher adherence to the TLD had higher odds of metabolic health (OR: 1.83, 95% CI: 1.09–3.91).^(^ ^75^ ^)^ Among Lebanese older adults aged over 50 years, compared to other dietary patterns prevalent among the study participants, the TLD showed the highest correlations with diet quality indices such as the Diet Diversity Score, the alternate Healthy Eating Index (aHEI), and the DASH-stye Diet score.^(^ ^76^ ^)^ Among preschool children, a national study showed that those with the highest adherence to the TLD pattern have 67% lower odds of being overweight or obese, compared to those with the lowest adherence to this dietary pattern (OR 0.33; 95% CI 0.11, 0.97).^(^ ^73^ ^)^ On the other hand, adherence to the Western type of diet showed significant associations with obesity, hyperglycemia, and the metabolic syndrome among Lebanese adults and adolescents.^(^ ^70^ ^,^ ^77^ ^,^ ^78^ ^)^ While no single country or definition of the Mediterranean diet exits, until 2015, all the available versions of the Mediterranean diet belonged to the West side of the basin, to countries such as Italy, Spain, Greece, Crete, and France.

The geographical location of Lebanon as a Mediterranean country, the composition its traditional dietary pattern as well as the positive health associations of this traditional dietary pattern with several health aspects led, in 2015, to proposing this diet as a version of what is known as the Mediterranean diet.^(^ ^79^ ^)^ Using national dietary data, Naja *et al.* ^(^ ^80^ ^)^ developed a Lebanese Mediterranean Diet (LMD) index, comprised of nine key food groups; fruits, vegetables, legumes, olive oil, burghol, dairy products, starchy vegetables, dried fruits, and eggs. Belonging to the third tertile of the dietary intake of these foods/food groups corresponded to a ‘3’ point on the score, while belonging to the first tertile corresponded to 1 point of the score. As such, the score of the TLD ranged between 9 and 27, with higher values indicating a greater adherence to this dietary pattern. The scores of the TLD were positively associated with other Mediterranean diet scores from Greece, Italy, Spain, and France with the highest correlation being with the Italian Mediterranean diet.^(^ ^80^ ^)^ Positioning the Lebanese diet as an eastern Mediterranean variant can enrich Mediterranean diet definitions and complements research on its diversity and health impact.

Following the release of the SDGs and growing recognition of the Mediterranean diet as sustainable and healthy, dietary guidelines were revised to include sustainability, human health, and social acceptability. Notably, countries that have incorporated these criteria include Germany, Brazil, Sweden, Qatar, as well as the Nordic countries that include Denmark, Finland, Iceland, Norway, and Sweden. These updates reflect a growing trend towards integrating planetary health into national dietary guidance. In this context, a national examination of the environmental footprints (EFP) of the dietary patterns prevalent in Lebanon was conducted. The environmental footprints considered were water use, energy use, and GHG emissions which were calculated using a review of available life cycle analyses. Specifically, the three dietary patterns identified were the Traditional Lebanese, high protein and Western.^(^ ^72^ ^)^ Among these three dietary patterns, the TLD had the lowest water use and GHG per 1000 Kcal (Water (L/Kg): 443.61 ± 197.15, 243.35 ± 112.0, 264.72 ± 161.67; GHG (KG CO2 eq/day) 0.58 ± 0.32, 0.38 ± 0.24, 0.57 ± 0.37, for the Western, TLD and High-Protein, respectively). The scores of the TLD pattern were associated with lower odds of energy use, whereas those of the high-protein dietary pattern were associated with higher odds of the three EFPs.^(^ ^72^ ^)^ Since late 2019, Lebanon has faced a cascading series of crises, including detrimental economic, health and socio-political challenges, the COVID-19 pandemic, and the 2020 Beirut port explosion. These challenges took place while the country continues to struggle with a heavy burden of malnutrition and non-communicable diseases, all imposing serious repercussions on people’s livelihoods, food security, and health.^(^ ^81^ ^)^ Such circumstances have motivated a study to identify and characterise a Lebanese dietary pattern that also falls within the limits of three main constraints: human health, cost and environmental footprints.

In conclusion, TLD emerges as a culturally rooted, health-promoting, and environmentally sustainable dietary pattern. Its alignment with the broader Mediterranean diet adds valuable diversity to the concept, especially from the Eastern basin. Evidence consistently links adherence to the TLD with lower risk of overweight and obesity among preschool children (OR = 0.33; 95% CI: 0.11–0.97),^(^ ^73^ ^)^ better metabolic health among overweight and obese adults (OR = 1.83; 95% CI: 1.09–3.91),^(^ ^75^ ^)^ and higher diet quality scores among older adults.^(^ ^76^ ^)^ From an environmental perspective, TLD is associated with lower water use, energy use, and GHG emissions per 1,000 kcal consumed, compared with Western and High-Protein dietary patterns.^(^ ^72^ ^)^ These findings underscore its potential as a model dietary pattern that simultaneously supports public health, cultural identity, and planetary sustainability.

### African Indigenous foods as culturally rooted approaches to sustainable diet

In the African context, indigenous foods represent a critical but underutilised component of SFSs. These foods are not only ecologically adapted and nutritionally rich but also deeply rooted in local cultures and traditional knowledge systems.^(^ ^82^ ^)^ This case study explores the integration of African indigenous foods into sustainable diets, drawing from examples across sub-Saharan Africa including Ethiopia, Nigeria, Kenya, Malawi, South Africa, and Zimbabwe. It assesses the ecological, nutritional, and cultural significance of these foods and provides scientific evidence of their contributions to dietary sustainability.

### Indigenous foods and the sustainable diets framework

The FAO/Bioversity International Sustainable Diets Framework^(^ ^1^ ^)^ is multidimensional, encompassing health, environmental sustainability, affordability, and cultural acceptability. Indigenous foods, defined as plant and animal species that are native or have long been part of traditional African food systems,^(^ ^83^ ^)^ align well with this framework. These foods include traditional grains such as sorghum (*Sorghum bicolor*), millet (*Pennisetum glaucum*), and teff (*Eragrostis tef*), legumes such as bambara groundnut (*Vigna subterranea*); root crops like yam (*Dioscorea spp.*), and leafy vegetables such as amaranth (*Amaranthus spp.*), cowpea leaves (*Vigna unguiculata*), and African nightshade (*Solanum scabrum*). Indigenous African foods have often been displaced by colonial-era and Green Revolution policies that favoured monoculture crops like maize and wheat.^(^ ^84^ ^,^ ^85^ ^)^ However, emerging research shows that traditional foods can support more resilient, equitable, and healthy food systems, especially under conditions of climate variability,^(^ ^86^ ^)^ land degradation, and changing food consumption patterns.

For example, African indigenous foods (food crops. wild harvested fruits and vegetables, and wild meat) contribute to sustainable diets through various pathways. Nutritionally, these foods are rich in micronutrients that are often lacking in modern diets dominated by refined grains and imported vegetables. For instance, indigenous vegetables such as amaranth and nightshade contain significantly higher levels of iron and provitamin A carotenoids than cabbage or lettuce.^(^ ^87^ ^,^ ^88^ ^)^ Environmentally, indigenous crops are adapted to local climatic and soil conditions, requiring fewer external inputs such as synthetic fertilisers and irrigation water.^(^ ^89^ ^,^ ^90^ ^)^ This reduces their environmental footprint and supports ecosystem services such as soil conservation and biodiversity maintenance. Socially and culturally, indigenous foods support food sovereignty, intergenerational knowledge transfer, and community identity.^(^ ^91^ ^)^ They are often grown and prepared by women, who serve as custodians of traditional agricultural and culinary knowledge. Supporting indigenous food systems thus contributes to gender empowerment and community resilience. Moreover, these foods help buffer households against shocks such as droughts, supply chain disruptions, and price volatility, factors that are increasingly common under climate change.^(^ ^88^ ^)^

### Traditional grains in the Sahel and horn of Africa

In the arid and semi-arid zones of the Sahel and the Horn of Africa, traditional cereals such as sorghum, millet, and teff are dietary staples that form the basis of SFSs. In Ethiopia, teff is the primary ingredient in *injera*, a fermented flatbread that is central to Ethiopian cuisine. Teff is rich in fibre, calcium, iron, and resistant starch, and is naturally gluten-free, making it a nutritionally superior alternative to many imported grains.^(^ ^92^ ^)^ In Niger and Mali, millet and sorghum have sustained rural communities for centuries and are prized for their drought tolerance, requiring far less water than maize or rice.^(^ ^93^ ^)^ These crops also tolerate poor soils, are grown with minimal external inputs, and contribute to agro-biodiversity. Their continued cultivation supports climate resilience, while their consumption helps preserve culinary traditions and local food sovereignty.

### Leafy vegetables in East and Southern Africa

African Indigenous Vegetables (AIVs), which are plant species, both wild and domesticated, that originate from Africa or have been cultivated in African agro-ecological zones for centuries, forming part of the continent’s traditional food systems,^(^ ^94^ ^)^ are prominent in East and Southern Africa, where they are consumed regularly in both rural and peri-urban households. AIVs are characterised by their long history of local use, cultural significance, and adaptation to local climates, soils, and farming practices. These vegetables are often grown in home gardens and collected from the wild, embodying both ecological knowledge and culinary tradition.^(^ ^95^ ^)^ In Kenya and Zimbabwe, vegetables such as spider plant (*Cleome gynandra*), amaranth, and African nightshade are integral to local diets and are rich sources of iron, calcium, folate, vitamin A, and antioxidants.^(^ ^88^ ^)^ The African Leafy Vegetables Project in Kenya has successfully raised awareness and market access for these vegetables, resulting in increased agricultural production, dietary diversity, and incomes among smallholder women farmers.^(^ ^96^ ^)^ In Malawi, cowpea leaves are commonly consumed and incorporated into school feeding programmes to enhance micronutrient intake among children.^(^ ^97^ ^)^ Research by Uyoga*et al.* ^(^ ^98^ ^)^ demonstrated that incorporating AIVs into children’s meals significantly improved their vitamin A and iron status. South Africa and Zimbabwe also presents a culturally rich case, where dishes such as *morogo*, a mix of amaranth, spider plant, and blackjack (*Bidens pilosa*), are traditionally prepared and valued for both their taste and cultural symbolism.^(^ ^98^ ^,^ ^99^ ^)^

### Legumes and roots in West Africa

In West Africa, indigenous legumes such as bambara groundnut and African yam bean are gaining renewed attention. Bambara groundnut is highly drought-tolerant and nutritionally dense, with 15–20% protein content; specifically with high levels of lysine and methionine, amino acids often deficient in staple cereals.^(^ ^100^ ^)^ The crop also improves soil fertility through nitrogen fixation and grows in marginal soils where other legumes struggle.^(^ ^101^ ^)^ African yam bean has similarly been used in subsistence farming systems to enhance protein intake and diversify cropping patterns. Yam and cassava are deeply engrained in the food cultures of Nigeria, Ghana, and Côte d’Ivoire. Yams, in particular, play important roles in festivals and life-cycle ceremonies, linking food systems to social structures and cultural meaning.^(^ ^102^ ^,^ ^103^ ^)^ These root crops provide significant caloric intake and are often prepared in ways that reflect regional identities, such as *pounded yam* or *fufu*. Their integration into sustainable diets ensures not only nutrient security but also cultural continuity. In summary, African indigenous foods in general, provide a compelling model for sustainable diets that are not only nutritionally adequate and environmentally sound, but also socially and culturally meaningful. Their promotion and continued integration into modern food systems require a holistic approach that values cultural identity, supports biodiversity, and enhances resilience in the face of climate change. As Africa seeks to achieve greater food security, improved nutrition, and environmental sustainability, indigenous food systems must be placed at the centre of the sustainable diets agenda.

## Policy and governance

As the body of evidence for associations between dietary patterns and health and sustainability outcomes has grown, there has been an increasing number of calls for the United Nations and national governments to develop policy responses to promote healthy and sustainable diets. It is nearly 40 years since Gussow and Clancy published their landmark paper arguing the case for ‘Sustainable dietary guidelines’.^(^ ^52^ ^)^ In the intervening period, 37 national governments have heeded this argument and officially recognised the importance of incorporating a sustainability dimension into their national dietary guidelines, albeit usually to a modest extent.^(^ ^34^ ^)^ The publication of the FAO and WHO’s ‘*Sustainable healthy diets – Guiding principles*’^(^ ^104^ ^)^ has provided a comprehensive report to help support more governments to view their future revisions of national dietary guidelines through a sustainability lens. The recommendations outlined in these international and national nutrition policy reference standards are broadly consistent and can be captured in three core diet principles that integrate health and sustainability considerations^(^ ^29^ ^)^:

- ‘Variety – to help achieve a nutritionally adequate diet and help protect the biodiversity of food systems.
- Balance – to help reduce risk of diet-related non-communicable diseases and excessive use of finite environmental resources and production of GHG emissions.
- Moderation – to help achieve a healthy body weight and avoid wasting finite environmental resources used in providing food surplus to nutritional requirements’.

Many scientific challenges still need to be tackled to encourage the further development of evidence-informed nutrition policy reference standards. Current evidence synthesis methods, rooted in the 1990s evidence-based medicine movement, rely on a hierarchy of evidence that rates study quality by its control of internal bias.^(^ ^105^ ^)^ While effective for assessing nutrient–health associations in clinical settings, these methods are less suited to capturing the complex, context-specific links between dietary patterns, health, and sustainability.^(^ ^106^ ^)^ Scientists recommend complementing current methods with approaches that incorporate Indigenous knowledge on the health and sustainability of traditional diets.^(^ ^107^ ^)^

Regardless of the quantity and quality of the evidence base informing nutrition policy reference standards, they need to be translated into food and nutrition policy actions to promote healthy and sustainable diets across populations and healthy and SFSs to support those diets. However, people generally select foods and not whole diets. Correspondingly, food and nutrition policy actions commonly are directed at food labelling, food taxation, food marketing and food procurement initiatives. So, the challenge for nutrition policymakers and practitioners is how to translate dietary-level recommendations into food-level healthiness and sustainability advice. Therefore, policy tools for promoting sustainable diets extend beyond nutrient profiling systems and include fiscal measures such as taxation, regulation of food marketing (particularly to children), and sustainable public procurement programmes in schools, hospitals, and other institutions.^(^ ^108^ ^,^ ^109^ ^)^

The most prominent metrics used globally to assess a food’s ‘healthiness’ and ‘sustainability’ are nutrient profiling models, e.g., the Australasian Health Star Rating (HSR) system,^(^ ^110^ ^)^ and life cycle assessment, e.g., estimates of GHG emissions for individual foods,^(^ ^111^ ^)^ respectively. The use of these reductionist, i.e., reducing complex and context-rich dietary pattern exposures to a limited selection of isolated nutrients and reducing complex and context-rich food system dynamics into discrete disconnected food supply chain components, approaches to assess complex dietary pattern impacts on health and sustainability outcomes is associated with problematic assumptions and validity concerns.^(^ ^6^ ^,^ ^112^ ^,^ ^113^ ^)^ For example, an evaluation of the HSR system reported it was providing a ‘health halo’ for almost 75% of ultra-processed foods displaying health stars, despite these foods being unhealthy and unsustainable.^(^ ^114^ ^)^ In addition, there is a lack of evidence that food-related life cycle assessments can capture the complexity and context-richness of food system dynamics and their multiple environmental impacts to assess the sustainability of individual foods. A simple food healthiness and sustainability rating system based on the Nova food processing classification system would provide a more conceptually and theoretically authentic approach for designing food and nutrition policy actions.^(^ ^29^ ^)^ These conceptual and theoretical underpinnings to the Nova classification system and how it is aligned with sustainable diets are supported by a large and growing body of evidence.^(^ ^7^ ^–^ ^10^ ^,^ ^28^ ^)^ This body of evidence is being translated into official healthy and sustainable dietary guidance with UPF-related recommendations included in international^(^ ^115^ ^)^ and national (Brazil) dietary guideline reports. Therefore, the Nova classification system provides a particularly robust solution for translating evidence of sustainable diets into a metric for assessing an individual food’s sustainability based on whether it is categorised as Nova food group 1-3 or Nova food group 4 (ultra-processed food).

Food systems have been described as ‘entry points’ for acting on food and nutrition policy reports.^(^ ^69^ ^)^ They extend across food production, processing, trade, financing, transport, storage, selling, marketing, consumption, and disposal. Transforming food systems to promote population and planetary health requires a variety of food and nutrition policies that collectively engage with each of these food system components. Specific policies are the responsibility of different levels (international, national, state) of government and different sectors (agriculture, finance, trade, industry, education, health, etc.) within governments. A comprehensive and coherent national food and nutrition policy is needed to efficiently and effectively plan, implement and evaluate the variety of potential policy actions across different levels and sectors of government.^(^ ^116^ ^)^

## Knowledge gaps and future research

Despite the growing momentum in promoting sustainable diets, several knowledge gaps constrain their operationalisation, particularly in diverse sociocultural and agroecological contexts. A key limitation is the lack of consensus on standard metrics to assess the sustainability of diets across the environmental, health, social, and economic domains. While tools such as the Sustainable Diet Index^(^ ^18^ ^)^ and Life Cycle Assessment (LCA) have been developed,^(^ ^117^ ^)^ they are often limited in their integration of all sustainability dimensions or adapted only to high-income country contexts. One critical gap is the contextualisation of sustainability assessments to low- and middle-income countries (LMICs). Most evidence on the environmental impacts of dietary patterns is derived from high-income settings, using aggregated global datasets that often overlook subsistence agriculture, informal markets, and food culture diversity.^(^ ^118^ ^)^ There is a need to expand empirical studies on the sustainability of diets in LMICs using locally relevant indicators that integrate traditional knowledge, ecological zones, and social dynamics.

Another under-researched area is the triple burden of malnutrition and its relationship with food system sustainability. The triple burden of malnutrition, which refers to the simultaneous presence of undernutrition, micronutrient deficiencies, and overweight/obesity with related non-communicable diseases (NCDs), is a major global health challenge affecting populations across the socioeconomic spectrum.^(^ ^119^ ^)^ Although sustainable diets aim to address both over- and under-nutrition,^(^ ^120^ ^)^ empirical evidence on how dietary shifts affect micronutrient adequacy, especially in nutritionally vulnerable populations such as women, children, and the elderly, remains limited. The promotion of plant-based diets, for instance, needs to be accompanied by strategies to ensure bioavailability of key micronutrients such as iron, calcium, and vitamin B12.^(^ ^21^ ^)^ Behavioural and sociocultural drivers of dietary choices are often inadequately considered in sustainability research. Studies have shown that food preferences, perceptions of healthiness, cultural taboos, and gender norms shape food consumption more than environmental or health concerns alone.^(^ ^121^ ^,^ ^122^ ^)^ There is thus a need for greater interdisciplinary research that incorporates behavioural sciences, anthropology, and participatory approaches to design interventions that are locally acceptable and scalable. Improving the food environment will require not only multisectoral nutrition interventions but also strengthening the legal and regulatory frameworks that govern food production, labelling, marketing, and trade.^(^ ^123^ ^)^ Such measures can help shift consumption patterns towards healthier and more sustainable diets.

The interaction between food system transformation and climate resilience, encompassing synergies, trade-offs, equity impacts, and policy integration, remains critical. The interaction between food system transformation and climate resilience is also insufficiently explored. With increasing climate variability, the sustainability of diets must be analysed not only in terms of mitigation (e.g., GHG reduction) but also in terms of adaptation and resilience (e.g., dietary diversity, seasonality, and local crop systems). Studies that link climate-smart agriculture to sustainable diet outcomes remain sparse. While environmental sustainability is well-researched, few studies comprehensively evaluate the cost and economic access to sustainable diets. The recent work by Hirvonen *et al.* ^(^ ^124^ ^)^ showed that more than three billion people globally cannot afford healthy diets as recommended by the EAT-Lancet Commission. Future research should focus on developing locally tailored, affordable diet scenarios that meet both nutritional and sustainability criteria.

Finally, governance mechanisms and policy coherence across sectors, agriculture, trade, health, environment, are poorly documented. Research should explore policy innovations, public procurement, fiscal tools, and institutional interventions that create enabling environments for sustainable diets. Additionally, the incorporation of implementation science is needed to evaluate the real-world effectiveness and scalability of interventions aimed at promoting sustainable diets in different contexts. In summary, advancing research on sustainable diets demands multidimensional, context-specific, and transdisciplinary approaches. Building local evidence bases, harmonising sustainability metrics, and co-developing interventions with communities and policymakers will be critical for translating the global vision of sustainable diets into locally actionable realities.

## Discussion and Conclusion

The urgent need for more SFSs has brought sustainable diets to the forefront of global health and environmental discourse. This manuscript has explored the scientific advances, practical case studies, and policy implications of sustainable diets, revealing both promising pathways and persistent challenges. The evidence strongly supports the role of plant-forward diets, increased consumption of whole and minimally processed foods, and reduced reliance on industrialised animal agriculture as crucial levers to improve health outcomes while mitigating environmental degradation.^(^ ^12^ ^,^ ^13^ ^)^ However, translating the science of sustainability into everyday dietary practice will require more than technical models of the diet of the future. The diets of human beings are as social, cultural, and economic as they are physical. Translating sustainability science into lived dietary practices requires more than technical fixes or universal dietary models. As the examples provided demonstrate, the impacts of sustainable diets will depend on connecting sustainable eating patterns to regional, local food cultures, purchasing power, availability, and knowledge. Food consumption patterns based on traditional and indigenous food systems, particularly in African and Asian communities, offer promising examples that are nutrient-rich, biodiversity-friendly, and culturally based.^(^ ^118^ ^)^ These findings affirm that sustainable diets are not a singular prescription, but a plural and dynamic set of pathways tailored to ecological and cultural diversity. On the scientific front, there is still room for improvement in the identification of better indicators able to represent the multiple dimensions of diet sustainability, taking into account environmental but also nutritional, cultural, and economic aspects. Equally important is generating disaggregated ranges of data, so that shifts in diets do not exacerbate existing inequalities. The inclusion of community voices and experiences in the design of food policy and research is essential for relevance, legitimacy, and sustainability.

Therefore, there is a need to consider ecological and cultural diversity in the design of sustainable diets a plurality and dynamics of pathways is particularly indispensable. However, if the exclusive structures and practices in food systems are not addressed in a real way, then the fine, ambitious, beautiful story of a SFS may mean that we remain invisible and excluded Without addressing the structural inequalities in food systems, there is still a risk of further marginalising vulnerable populations in the context of sustainable diets. For example, promoting low-emission diets in communities facing food insecurity must be carefully balanced with nutritional adequacy, particularly for children, pregnant women, and those with micronutrient deficiencies.^(^ ^124^ ^)^ Thus, sustainability must be pursued in tandem with equity and food sovereignty. This is made increasingly possible by the fusion of governance and policy. Although more and more common, the integration of sustainability aspects in national FBDGs, public procurement programs, school meal programs, and food labelling legislation necessitate political commitment, intersectoral coherence, and participatory processes.^(^ ^20^ ^)^ Governments, civil society, academia, and the private sector must co-create solutions that support local food economies, invest in nutrition-sensitive agriculture, and foster behaviour change through education and incentives.

In conclusion, sustainable diets represent a promising, yet complex, pathway towards transforming food systems to support both planetary and human health. They are not merely about what individuals choose to eat but are a reflection of systemic interactions between policy, culture, economy, and environment. Realising their full potential will require a holistic and inclusive approach, one that champions diversity, prioritises the most vulnerable, and recognises the centrality of food to human identity, dignity, and resilience.
